# Risk factors associated with low handgrip strength in the older Korean population

**DOI:** 10.1371/journal.pone.0214612

**Published:** 2019-03-28

**Authors:** Chung Reen Kim, Young-Jee Jeon, Taeheum Jeong

**Affiliations:** 1 Department of Physical Medicine and Rehabilitation, Ulsan University Hospital, University of Ulsan College of Medicine, Ulsan, South Korea; 2 Department of Family Medicine, Ulsan University Hospital, University of Ulsan College of Medicine, Ulsan, South Korea; Ehime University Graduate School of Medicine, JAPAN

## Abstract

Hand grip strength (HGS) is a means to assess health status and physical abilities indirectly. Various factors are known to be related to HGS, but data on the factors influencing HGS in the Korean population are lacking. Recently, the Korea National Health and Nutrition Examination Survey (KNHANES) conducted by the Korea Centers for Disease Control and Prevention surveyed social status, nutrition, physical status, and other medical history including HGS. This study aimed to investigate the social, physical, and individual health behavior factors associated with low HGS in the elderly Korean population. This population-based cross-sectional study was based on the 2014–2016 KNHANES. Community-dwelling Korean elderly (aged ≥65 years) were included in this survey. The relationship between HGS and socioeconomic status, nutrition, exercise, and other clinical characteristics were analyzed using multivariate logistic regression. The sample size was 3,634 (weighted: 6,006,406). Elderly men and women with low HGS showed lower body mass index, reduced physical activity and lower education level. Among elderly men, excessive carbohydrate and inadequate protein intake were observed in the low HGS group. Meanwhile, older women who did not drink alcohol and had diabetes were at higher risk of low HGS. In conclusion, various physical, nutritional, socioeconomic and comorbidity statuses were seemed to be the factors associated with HGS in Korean elderly. However, further cohort or case-control study will be necessary to determine the causal relationship of those factors and HGS.

## Introduction

Sarcopenia, the age-related loss of skeletal muscle mass and function, is associated with poor quality of life, disability and mortality [1–3]. Because South Korea is one of the fastest aging countries in the world, the prevalence of sarcopenia is also rapidly increasing [4]. Recently, sarcopenia has become one of the most serious public health issues in Korea. Accordingly, there has been increased interest and related research on sarcopenia [5].

The prevention and treatment of sarcopenia begins with the identification of individuals at risk of the condition. The diagnosis of sarcopenia is based on the assessment of muscle mass, muscle strength, and physical performance. Recently, the European Working Group on Sarcopenia in Older People and the Asian Working Group of Sarcopenia suggested cut-off values for evaluation of muscle mass, muscle strength, and physical performance [6, 7]. Among the measures of muscle strength, handgrip strength (HGS) is widely used because it can be easily measured in the clinical setting [8]. Previous studies have revealed that low HGS is associated with falls, disability, impaired health-related quality of life, and increased mortality [9–11]. Also, various factors related to HGS have been identified. Although old age, low body mass index (BMI), and less physical activity was highly related to lower HGS, the relationship between HGS and other factors such as marital status, level of education, health related behaviors and comorbidities showed inconsistent results [12–14].

However, there is still a lack of studies on HGS in the elderly Korean population. Quan et al. previously examined the relationship between HGS and physical ability, socioeconomic status, health behaviors, and comorbidities [15]. However, there were limitations that only male subjects were included and the number of subjects was small. Recently, large scale data of HGS were provided for the Korean population by the Korea National Health and Nutrition Examination Survey (KNHANES). Based on these data, normative reference values of HGS of Koreans have been recently established, and cut-off values for low HGS were established (28.9 kg for men and 16.8 kg for women) [16]. In addition to HGS, The KNHANES also provided the information on physical, socioeconomic, nutritional, health behavior, and comorbidity status of the elderly population, and it is now possible to identify the association of HGS and relevant factors in Korean elderly. Therefore, in the present study, we aimed to investigate the factors related to low HGS in the Korean elderly population using KHNANES data from 2014–2016. Based on the results of this study, we hope that it will serve as a guide for further interventional studies about HGS and also help to orient policy to improve Korean elderly with sarcopenia.

## Materials and methods

### Data source and study participants

This study used data from the KNHANES conducted by the Korea Centers for Disease Control and Prevention from 2014–2016. KNHANES is an annually conducted nationwide, population-based health survey, and its target population is non-institutionalised civilians in the Republic of Korea. The sample frame was determined based on the population and housing census, and the representative households were selected using a stratified multistage clustered probability sampling design. Annually 192 enumeration districts were selected each year, and 20 households in each enumeration district were chosen by a rolling sampling system to represent all parts of the nation. The 2014–2016 KNHANES assessed the health and nutritional statuses of 23,080 Koreans. Among these, 3,634 individuals (1,692 men and 1,942 women) who were aged 65 years or older and completed both a health interview and examination including measurement of HGS were identified as study participants. Because the KNHANES is a weighted survey, 3,634 participants represented a total of 6,006,406 participants.

The KNHANES is composed of three component surveys: a health interview, a health examination, and a nutrition survey. Trained medical staff and interviewers performed the health interviews and examinations. Health behaviors such as cigarette smoking, alcohol use, and physical activity were collected via self-report. Information about medical conditions, education, and income were identified via face-to-face interviews. Health examinations consisted of anthropometric measures such as height, weight, blood pressure, and biochemical profile using fasting blood serum and urine.

Each participant provided informed consent prior to inclusion in the study. KNHANES in 2014 was approved by the Korea Centers for Disease Control and Prevention institutional review board (2013-1212EXP-03-5C, 2015-01-02-6C). However, in accordance with the Korean bioethics law, institutional review board approval for KNHANES in 2015–2016 was waived because it was conducted by the Korean government for public welfare.

### Measurement of hand grip strength

The 2014–2016 KNHANES measured HGS among participants aged ≥10 years. HGS was not measured for participants with no hands, arms, or thumbs; hand paralysis; cast in hands or fingers; bandaged hands or wrist/hands; wrist surgery in the recent 3 months; and pain, tingling, or stiffness in the hands or wrist within the preceding week.

HGS was measured using a digital grip strength dynamometer (TKK 5401 GRIP D; Takei, Japan), which measures 5.0–100.0 kg of force and has an adjustable grip span. Participants performed three trials for each hand alternatively, always starting with the dominant hand. Participants were instructed to squeeze the grip continuously with full force for at least 3 seconds. The average of three trials for each hand was recorded. The average of three trials for each hand was recorded. Low HGS was defined according to a previous study [16]. The cut-off value was <28.9 kg for men and <16.8 kg for women.

### Description of variables

#### Lifestyle and physical activity

Drinking, smoking, and physical activity statuses were assessed via self-report [16]. Participants who performed aerobic exercise were identified as those who spent >150 minutes on moderate physical activity or 75 minutes on high-intensity physical activity per week. Participants who performed strengthening exercise were identified as those who performed strengthening exercise >once weekly.

#### Nutritional status

The nutrition survey in KNHANES addresses dietary behaviors, food frequency, and food intake and is conducted through a face-to-face interview. The food intake questionnaire was designed as an open-ended survey for reporting various dishes and foods consumed using the 24-hour recall method. The amounts of total energy and other macronutrient intake were calculated by referencing the nutrient contents of foods described in the Korean Food Composition Table [17, 18].

To evaluate inadequate or excessive intake of nutrients according to age and sex, we used the dietary reference intakes for Koreans 2015 [19]. Nutrient intakes were compared to the estimated average requirements for total energy intake (1600 kcal/day for women, 2000 kcal/day for men) and protein (40 g/day for women, 45 g/day for men). For carbohydrate and fat intake, the acceptable macronutrient distribution range (ADMR) was used. Excessive carbohydrate intake was defined as > 65% of the ADMR, and excess fat intake was defined as > 30% of the ADMR.

#### Socioeconomic status

Monthly household income and education were considered as the main indicators of socioeconomic status. Regarding education, the participants were asked about their highest degree of educational attainment. This was classified into three educational categories: completion of elementary school (≤6 years), middle (7–9 years) and high school (10–12 years), and post-secondary school (≥13 years). Household income was calculated based on equivalent income (i.e., total household income divided by the square root of the number of household members) and classified into quartiles.

#### Anthropometric and comorbidities

BMI was calculated by dividing the weight in kilograms by the square of the height in meters. We identified chronic diseases such as hypertension, diabetes, stroke, ischemic heart disease, and chronic obstructive pulmonary disease (COPD). Hypertension was defined as one of the following: systolic blood pressure ≥140 mmHg and/or diastolic blood pressure ≥90 mmHg; receiving antihypertensive treatment; or an answer of “yes” to the question, “Have you ever been diagnosed with hypertension by a physician?”. Diabetes was defined as one of the following: fasting glucose ≥126 mg/dL; use of oral hypoglycemic agents or insulin therapy; or an answer of “yes” to the question, “Have you ever been diagnosed with diabetes by a physician?”. Arthritis, ischemic heart disease, and COPD were defined as an answer of “yes” to the question, Participants who had dental problems were also identified based on the question “Do you have any difficulty in chewing food due to dental problems such as teeth, dentures, and gums?”

### Statistical analysis

The characteristics of the study participants were presented as means (standard error) or percentages (standard error) as appropriate. Multivariate logistic regression was used to examine the odds of low HGS for each related factor with adjustment for age (Model 1). Next, we performed an additional analysis by adjusting the variables that were statistically significant in Model 1 (Model 2). All data were analyzed separately for men and women. The analysis reflects a complex survey sample design, which includes stratification, clustering, and weighting. All analyses were performed using SPSS version 22.0 (SPSS Inc., Chicago, IL, USA), and p<0.05 was considered significant.

## Results

The mean age of the male and female participants was 72.4 and 73.0 years, respectively. The mean BMI was 23.6 kg/m^2^ for men and 24.4 kg/m^2^ for women. The mean HGS was 31.6 kg for men and 19.0 kg for women, and 32.0% of men and 33.2% of women had low HGS. The detailed participant characteristics are shown in Table 1.

**Table 1:** Characteristics of participants (n = 3,634, N = 6.0x10^6^).

	Male (n = 1,692, N = 2.9x10^6^)	Female (n = 1,942, N = 3.1x10^6^)
**Age (years)**	72.4 (0.1)	73.0 (0.1)
**Body mass index (kg/m**^**2**^**)**	23.6 (0.1)	24.4 (0.1)
**Hand grasp strength, Dominant hand (kg)**	31.6 (0.2)	19.0 (0.1)
**Low Hand grasp power (%)**	32.0 (1.4)	33.2 (1.3)
**Alcohol drinking (%)**		
** No**	9.9 (0.8)	39.5 (1.2)
** Yes**	90.1 (0.8)	60.5 (1.2)
**Smoking status (%)**		
** Never smoker**	20.0 (1.1)	92.9 (0.7)
** Ex-smoker**	60.3 (1.4)	3.9 (0.5)
** Current smoker**	19.8 (1.2)	3.2 (0.6)
**Exercise (%)**		
** Aerobic exercise**	45.4 (1.5)	30.2 (1.3)
** Strengthening**	29.0 (1.3)	9.5 (0.8)
**Nutritional status (%)**		
** Inadequate protein intake**	29.6 (1.3)	46.9 (1.2)
** Inadequate energy intake**	59.8 (1.4)	67.2 (1.1)
** Excessive carbohydrate intake**	69.8 (1.2)	81.8 (1.0)
** Excessive fat intake**	2.4 (0.4)	3.1 (0.4)
**House income quartile (%)**		
** 1 (lowest)**	41.5 (1.5)	52.0 (1.4)
** 2**	28.2 (1.2)	24.0 (1.0)
** 3**	17.5 (1.1)	14.4 (0.9)
** 4 (highest)**	12.7 (1.1)	9.5 (0.9)
**Education level (%)**		
** ≤ 6 years**	41.7 (1.5)	75.5 (1.2)
** 7–9 years**	17.0 (1.1)	10.3 (0.8)
** 10–12 years**	25.0 (1.3)	10.1 (0.9)
** ≥ 13 years**	16.2 (1.2)	4.2 (0.6)
**Comorbidity (%)**		
** Hypertension**	58.4 (1.4)	66.6 (1.2)
** Diabetes**	24.5 (1.2)	23.7 (1.2)
** Arthritis**	13.9 (0.9)	44.5 (1.2)
** Ischemic heart disease**	9.1 (0.8)	7.0 (0.7)
** COPD**	2.3 (0.5)	0.8 (0.2)
**Dental problem (%)**	42.2 (1.4)	46.2 (1.2)

Values are reported as mean (standard error) or % (standard error).

COPD, chronic obstructive pulmonary disease

Compared with the participants in the normal HGS group, both male and female participants with low HGS were older and had lower BMI (Table 2). Moreover, both sexes in the low HGS group drank less and performed less aerobic and strengthening exercise. Also, inadequate nutritional intake was observed more frequently in the low HGS group than in the normal HGS group except excessive fat intake. However, the number of participants with low socioeconomic status was higher in the low HGS group. Dental problems were more frequent in the low HGS group in both sexes, and diabetes was more prevalent in the low HGS group in women.

**Table 2:** Differences between normal and low hand grip strength.

	Men			Women		
	Normal HGS (n = 1,118, N = 2.0x10^6^)	Low HGS (n = 574, N = 0.9x10^6^)	p value	Normal HGS (n = 1,296, N = 2.1x10^6^)	Low HGS (n = 646, N = 1.0x10^6^)	p value
**Age (years)**	71.1 (0.2)	75.1 (0.2)	<0.001	71.6 (0.2)	75.2 (0.2)	<0.001
**Body mass index (kg/m**^**2**^**)**	24.0 (0.1)	22.9 (0.1)	<0.001	24.6 (0.1)	24.0 (0.2)	0.003
**Hand grasp strength, Dominant hand (kg)**	35.5 (0.2)	23.5 (0.2)	<0.001	21.8 (0.1)	13.2 (0.1)	<0.001
**Alcohol drinking (%)**			<0.001			<0.001
** No**	7.8 (0.8)	13.8 (1.7)		33.5 (1.7)	51.3 (2.3)	
** Yes**	92.2 (0.8)	86.2 (1.7)		66.5 (1.7)	48.7 (2.3)	
**Smoking status (%)**			0.823			0.021
** Never smoker**	20.1 (1.4)	21.1 (2.1)		94.8 (0.7)	90.8 (1.5)	
** Ex-smoker**	60.2 (1.8)	60.5 (2.6)		3.2 (0.6)	5.5 (1.0)	
** Current smoker**	19.7 (1.5)	18.4 (1.9)		2.1 (0.5)	3.7 (1.2)	
**Exercise (%)**						
** Aerobic exercise**	50.4 (1.8)	34.6 (2.6)	<0.001	33.6 (1.7)	20.4 (2.1)	<0.001
** Strengthening**	34.4 (1.8)	18.6 (1.9)	<0.001	12.6 (1.1)	4.2 (0.9)	<0.001
**Nutritional status (%)**						
** Inadequate protein intake**	23.1 (1.5)	41.2 (2.4)	<0.001	41.5 (1.6)	55.7 (2.3)	<0.001
** Inadequate energy intake**	54.7 (1.8)	68.7 (2.3)	<0.001	62.4 (1.6)	74.0 (2.0)	<0.001
** Excessive carbohydrate intake**	65.5 (1.6)	78.8 (1.8)	<0.001	79.7 (1.4)	82.8 (1.8)	0.171
** Excessive fat intake**	3.1 (0.6)	1.2 (0.4)	0.021	3.5 (0.6)	2.5 (0.7)	0.304
**House income quartile (%)**			<0.001			<0.001
** 1 (lowest)**	34.6 (1.7)	53.5 (2.6)		46.9 (1.8)	59.6 (2.3)	
** 2**	31.3 (1.6)	23.2 (2.0)		26.5 (1.4)	20.4 (1.7)	
** 3**	19.6 (1.4)	14.0 (1.7)		15.6 (1.2)	12.2 (1.7)	
** 4 (highest)**	14.5 (1.4)	9.3 (1.6)		11.0 (1.3)	7.8 (1.2)	
**Education level (%)**			<0.001			<0.001
** ≤ 6 years**	35.4 (1.8)	56.3 (2.6)		68.7 (1.7)	86.9 (1.7)	
** 7–9 years**	18.1 (1.4)	14.4 (1.8)		12.6 (1.2)	6.8 (1.1)	
** 10–12 years**	28.3 (1.7)	16.9 (1.8)		12.9 (1.2)	3.9 (1.1)	
** ≥ 13 years**	18.3 (1.5)	12.5 (2.0)		5.8 (0.9)	2.4 (0.8)	
**Comorbidity (%)**						
** Hypertension**	59.3 (1.8)	56.3 (2.3)	0.297	65.1 (1.6)	69.1 (2.4)	0.191
** Diabetes**	23.1 (1.5)	24.9 (2.2)	0.526	21.2 (1.5)	30.2 (2.7)	0.002
** Arthritis**	11.8 (1.2)	16.3 (1.6)	0.018	42.4 (1.7)	41.4 (2.4)	0.770
** Ischemic heart disease**	8.4 (1.0)	9.3 (1.4)	0.618	6.3 (0.8)	7.5 (1.3)	0.369
** COPD**	2.2 (0.5)	1.8 (0.7)	0.598	0.6 (0.2)	1.2 (0.5)	0.189
**Dental problem (%)**	36.6 (1.8)	48.4 (2.5)	<0.001	41.4 (1.6)	48.2 (2.6)	0.029

Values are reported as mean (standard error) or % (standard error).

COPD, chronic obstructive pulmonary disease

We also investigated the factors associated with low HGS after adjusting for age (Model 1). Next, we performed an additional analysis by adjusting the variables that were statistically significant in Model 1 (Model 2). The analysis was conducted separately for men and women. The results for men were as follows (Table 3). The BMI of the low HGS group was lower than that of the normal HGS group. Elderly men who performed appropriate exercise were less likely to have low HGS (odds ratio [OR], 0.67; 95% confidence interval [CI], 0.51–0.89 for aerobic exercise and OR, 0.72; 95% CI, 0.52–0.99 for strengthening exercise). Men with low HGS consumed excessive carbohydrate (OR, 1.57; 95% CI, 1.18–2.11) and inadequate protein (OR: 1.50; 95% CI, 1.05–2.15) than those with low HGS. In the low HGS group, the number of elderly men with ≥13 years of education was lower than that of those who had been educated for **≤**6 years (OR, 0.62; 95% CI, 0.39–0.98).

**Table 3:** Multivariate analysis of factors associated with hand grip strength in men.

	Model 1			Model 2		
	Odds Ratio	95% CI	p value	Odds Ratio	95% CI	p value
**Age**				1.16	1.13–1.20	<0.001
B**ody mass index**	0.91	0.87–0.95	<0.001	0.92	0.88–0.96	<0.001
**Alcohol drinking**	0.66	0.45–0.96	0.032	0.75	0.49–1.14	0.181
**Smoking status**			0.893			
** Current smoker**	1					
** Ex-smoker**	1.09	0.77–1.54				
** Never smoker**	1.05	0.71–1.55				
**Exercise**						
** Aerobic exercise**	0.64	0.79–0.84	0.001	0.67	0.51–0.89	0.006
** Strengthening exercise**	0.58	0.43–0.78	<0.001	0.72	0.52–0.99	0.044
**Nutritional status**						
** Inadequate protein intake**	1.89	1.45–2.47	<0.001	1.50	1.05–2.15	0.027
** Inadequate energy intake**	1.41	1.09–1.83	0.009	1.17	0.85–1.60	0.342
** Excessive carbohydrate intake**	1.78	1.38–2.28	<0.001	1.57	1.18–2.11	0.002
** Excessive fat intake**	0.45	0.19–1.10	0.079			
**House income quartile, %**			0.051			
** 1 (lowest)**	1					
** 2**	0.69	0.51–0.94				
** 3**	0.67	0.46–0.98				
** 4 (highest)**	0.67	0.41–1.09				
**Educational level**			<0.001			0.001
** ≤ 6 years**	1			1		
** 7–9 years**	0.60	0.42–0.86		0.63	0.43–0.92	
** 10–12 years**	0.39	0.28–0.54		0.50	0.35–0.71	
** ≥ 13 years**	0.45	0.29–0.69		0.62	0.39–0.98	
**Comorbidity**						
** Hypertension**	0.81	0.63–1.04	0.094			
** Diabetes**	1.24	0.90–1.71	0.190			
** Arthritis**	1.36	0.94–1.97	0.104			
** Ischemic heart disease**	0.93	0.60–1.43	0.725			
** COPD**	0.74	0.31–1.80	0.510			
**Dental problem**	1.47	1.13–1.90	0.004	1.16	0.88–1.53	0.289

CI, confidence interval; COPD, chronic obstructive pulmonary disease

Model 1 was adjusted for age.

Model 2 was adjusted for age, body mass index, alcohol drinking, dental problem, aerobic and strengthening exercise, inadequate energy, protein and excess carbohydrate intake, and educational level.

For elderly women, the results of multivariate analysis were as follows (Table 4). The BMI of the low HGS group was also lower than that of the normal HGS group. Elderly women who drank alcohol were more likely to have normal HGS than non-drinking women (OR, 0.61; 95% CI, 0.45–0.82). Older women who performed appropriate exercise were less likely to have low HGS (OR, 0.60; 95% CI, 0.43–0.83 for aerobic exercise and OR, 0.39; 95% CI, 0.22–0.68 for strengthening exercise). There was no difference in nutritional status between the normal HGS and low HGS groups. Among the socioeconomic indicators, only educational level was also significantly different among the women. Older women who had been educated for 7–9 years and 10–12 years had lower risk of low HGS than those who had been educated for ≤6 years (OR, 0.57; 95% CI, 0.34–0.94 for 7–9 years and OR, 0.32; 95% CI, 0.17–0.61 for 10–12 years). There were more elderly women with diabetes in the low HGS group than in the normal HGS group (OR, 1.53; 95% CI, 1.09–2.15).

**Table 4:** Multivariate analysis of factors associated with hand grip strength in women.

	Model 1			Model 2		
	Odds Ratio	95% CI	p value	Odds Ratio	95% CI	p value
**Age**				1.10	1.06–1.13	<0.001
B**ody mass index**	0.96	0.92–0.99	0.014	0.93	0.89–0.97	0.001
**Alcohol drinking**	0.58	0.45–0.73	<0.001	0.61	0.45–0.82	0.001
**Smoking status**			0.274			
** Current smoker**	1					
** Ex-smoker**	0.90	0.38–2.10				
** Never smoker**	0.65	0.32–1.31				
**Exercise**						
** Aerobic exercise**	0.63	0.47–0.84	0.002	0.60	0.43–0.83	0.002
** Strengthening exercise**	0.35	0.21–0.57	<0.001	0.39	0.22–0.68	0.001
**Nutritional status**						
** Inadequate protein intake**	1.34	1.06–1.69	0.015	1.00	0.68–1.48	0.998
** Inadequate energy intake**	1.34	1.04–1.71	0.021	1.20	0.82–1.77	0.353
** Excessive carbohydrate intake**	1.06	0.78–1.45	0.695			
** Excessive fat intake**	0.80	0.39–1.64	0.547			
**House income quartile, %**			0.315			
** 1 (lowest)**	1					
** 2**	0.81	0.61–1.08				
** 3**	0.80	0.54–1.20				
** 4 (highest)**	0.77	0.52–1.15				
**Educational level**			<0.001			0.001
** ≤ 6 years**	1			1		
** 7–9 years**	0.59	0.38–0.93		0.57	0.34–0.94	
** 10–12 years**	0.32	0.17–0.58		0.32	0.17–0.61	
** ≥ 13 years**	0.44	0.19–0.99		0.56	0.23–1.36	
**Comorbidity**						
** Hypertension**	0.93	0.69–1.24	0.600			
** Diabetes**	1.43	1.06–1.92	0.020	1.53	1.09–2.15	0.013
** Arthritis**	0.96	0.74–1.24	0.753			
** Ischemic heart disease**	0.99	0.64–1.53	0.949			
** COPD**	2.00	0.68–5.87	0.205			
**Dental problem**	1.15	0.89–1.48	0.296			

CI, confidence interval; COPD, chronic obstructive pulmonary disease

Model 1 was adjusted for age.

Model 2 was adjusted for age, body mass index, alcohol drinking, aerobic and strengthening exercise, inadequate energy, protein intake, diabetes, and educational level.

## Discussion

In this study, we analyzed the factors associated with HGS by comparing the low and normal HGS groups among men and women aged ≥ 65 years. Alcohol drinking, exercise, dietary pattern, education level, diabetes, and BMI as well as age were the factors related to low HGS in the multivariate analysis.

First, we investigated the relationship between HGS and health behaviors such as alcohol drinking and smoking. In this study, alcohol drinking was inversely related to low HGS in both sexes after adjusting for age (Model 1), and among women, drinking alcohol was still inversely associated with low HGS after adjusting for variables that were statistically significant in Model 1 (Model 2). Although excessive drinking was known to be associated with sarcopenia in Korean postmenopausal women [20], light to moderate alcohol drinking has also been reported to be effective in preventing muscle mass reduction [21]. Similar to our findings, in the large cohort study conducted in Europe, it was also reported that current alcohol drinking was associated with an increase in HGS.[18] Although further additional research in needed, those results might suggest the possible positive effects of alcohol drinking on HGS. However, in case of smoking, there was no significant difference between the low HGS and normal HGS groups in the multivariate analysis in both sexes. Smoking is known to have a negative effect on HGS [22]. However, several studies about factors related to HGS showed various results [12, 18, 23]. A previous Korean study based on data from the early 2000s also showed no correlation between smoking and HGS [15]. Recently, the rate of smoking has declined due to the effects of the nationwide smoking cessation program and increasing cigarette price, which started from 2015 in Korea [24]. The proportion of Korean smokers aged 60 years and older reduced from 25.5% in 2000 to 14.4% in 2017 [25]. It is possible that this decrease in smoking rate has reduced the influence of smoking on HGS. However, long-term studies might be needed to confirm that smoking affects health.

Low participation rates in aerobic and strengthening exercise were significantly associated with low HGS in both genders. Exercise has been emphasized as a key strategy for sarcopenia treatment, along with nutritional supplementation [26, 27], and our findings support the importance of exercise for the elderly in Korea. Although there was a difference in the exercise participation rates between the low and normal HGS groups, an interesting observation was that the participation rate was low in both the low and normal HGS groups. The proportion of older Koreans with adequate exercise was <50%; particularly, the participation rate in aerobic and strengthening exercise was only 30% and 10%, respectively, in women. A study by Coups and Ostroff showed that the incidence of active physical activity in patients aged ≥65 years with or without cancer was approximately 20% [28]. In another previous British study, only approximately 50% of participants were physically active in their leisure time [29]. Therefore, rather than emphasizing the need for exercise only in the low HGS group, it is more important to educate the entire elderly Korean population. Due to the cross-sectional nature of this study, it is not possible to explain the causal relationship between exercise and HGS; thus, further studies are needed. In Japan, several studies on community-based exercise programs have been conducted, including the aging population [27], and high adherence to exercise intervention was observed [30, 31]. It could be helpful to develop appropriate community-based exercise programs for the elderly in Korea. In particular, strengthening exercise was highly correlated with HGS among elderly women; thus, it is important to emphasize strengthening exercise in elderly women as well as aerobic exercise.

Aside from exercise, nutrition is also an important factor for sarcopenia management [26]. Dietary protein has been considered as a potential determinant of muscle function, as aging is often associated with a reduction in food intake [32]. Additionally, the associations of muscle mass and physical performance with essential amino acids, milk protein, leucine, β-hydroxy β-methylbutyrate, and vitamin D intake have been reported [33, 34]. In this study, the low HGS group among men showed inadequate protein intake as well as excessive carbohydrate intake. In elderly women, although there was no statistically significant difference in protein intake between the low HGS and normal HGS groups, the proportion of inadequate protein intake of both HGS groups was considerably high. Especially, the proportion of inadequate protein intake in women with normal HGS group was as high as that of men with low HGS. These results suggested that the current quality of nutrition of the Korean elderly population seemed to be poor. Through subsequent intervention studies, it will be urgently necessary to provide education and policy on proper nutrition in the elderly population [35].

Additionally, this study also found that lower education levels were associated with high ORs for low HGS among both older Korean men and women. Previous Korean studies in older individuals showed similar results [15]. However, the findings of studies in other countries were contrasting. A European study found no significant association between HGS and educational level [23], while studies in Brazil found associations between HGS and certain factors [12]. This difference is presumed to be due to differences in the classification criteria or socioeconomic environments between the studies. Although the impact of education on HGS is unclear, the subjects with higher educational level seemed to be more conscious of the importance of physical activity in maintaining health [20]. In contrast, people with lower educational level showed lower physical activity [24]. Also family activity habits and dietary habits differed according to educational level [22]. Therefore, it is possible that the physical activity or dietary pattern according to the education level may eventually be related to HGS. Besides, higher education attainment may increase access to knowledge on health, practice of more healthy behaviors, employment opportunities, and income, all of which affect health [15, 35], and may also affect HGS and muscle strength [12]. In Korea, the participants had limited opportunity for higher education during their younger years. Particularly, the opportunity is relatively much lower for women than that for men [36, 37], and approximately 70% of older women received ≤6 years of education [15, 36]. However, in the future the relationship between HGS and education is expected to be gradually less affected like Europe because of compulsory education and higher college entrance rates in Korea. Meanwhile, appropriate education and information on sarcopenia, such as optimal nutrition and exercise, should be provided to the current elderly with lower education level.

Previously, Quan et al. reported significant differences in income between the low and high HGS groups in Korea [15], and they postulated that low income can limit overall health and functional status among older individuals. By contrast, income was not a significant factor affecting HGS in our multivariate analysis. This difference is considered a selection bias resulting from the difference in the number of subjects. In our large-population survey, the low-income population was larger than that in the previous study. Additionally, previous study conducted in Europe reported that income has a small impact on HGS [38]. Income is generally related to early and mid-life wealth [23], and may not fully represent financial resources available at old age, particularly after retirement. For example, older individuals frequently have substantial wealth but little income, primarily from pensions [39]. However, unlike income, wealth is associated with health in older individuals [23]. Thus, it is necessary to have a more comprehensive approach to assess wealth rather than income. In the future, a worsening of this imbalance may become a serious problem in Korea as the older population rapidly grows, and the number of the poor older individuals is expected to increase gradually [40]. Therefore, along with the establishment of socioeconomic policies that can reduce the proportion of poor older individuals, welfare policies for health care, nutrition, and information provision for the poor older population are also necessary.

We also conducted an analysis of association between HGS and several comorbidities, including the dental problem. However, only diabetes in women was significantly associated with low HGS. Previous studies on the association between diabetes and HGS have not yet been clarified. Some studies reported relevance [41, 42], while others reported a lack of relevance [38, 43, 44]. Previous studies on the relationship between the other chronic diseases and HGS have also yielded varied conclusions [38, 41, 44]. A previous study of elderly male Koreans did not show any association between HGS and chronic diseases such as hypertension, diabetes, and dyslipidemia [15]. Because this study briefly analyzed the prevalence of the disease, it did not clearly explain the relationship between chronic disease and HGS. More studies are needed to further investigate the duration, severity, and medications of each chronic disease.

For the dental problem, chewing ability was known to be associated with sarcopenia [45], and the prevalence of sarcopenia in older outpatients visiting the dental clinic was relatively high [46]. However, in this study, presence of the dental problem was not significantly associated with HGS in multivariate analysis. Although the difference between groups was not significant, dental problems were common in about 50% of subjects in this study. It is well known that older individuals with tooth loss lose their masticatory function, thus interfering with regular eating and limiting the intake of difficult to chew foods such as vegetables, fruits, and meat [47]. Therefore, as with other risk factors, dental problems also seem to be needed for the elderly as a whole.

Finally, while analyzing the research and interpreting the meaning, we found that each factor was not independently associated with HGS, but there was a close interrelation among those factors. For example, lower education levels impaired access to and understanding of sarcopenia-related information about nutrition, dental health, and exercise. Education level was also associated with low-paying jobs or low wealth. Low wealth also led to insufficient nutrition and lack of time for exercise. Dental problems were also associated with nutrition and education level. Therefore, efforts aimed at correction should be made organically and holistically to improve sarcopenia [26, 48, 49].

This study has some limitations. First, we cannot explain the causal relationship since this study was cross-sectional in nature. Based on this study, a future prospective cohort study will enable us clearly understand the causal relationship between the factors and HGS. In addition, the factors related to HGS in this study will become clearer if interventional studies are conducted. Second, low HGS alone does not fully represent sarcopenia. Muscle mass and physical performance should also be considered, but those data were not collected in the current survey. However, HGS is also a well-known predictor of health status, muscular strength, nutritional status, and disability [26, 50]. Thus, although data on muscle mass and physical performance are lacking, our study collected adequate data to evaluate sarcopenia. Third, the HGS values may vary according to the equipment and method used for its assessment [8]. However, the cut-off value for HGS was assessed using the same equipment and method in the KNHANES and previous studies. Thus, the bias according to the evaluation method is considered to be small.

## Conclusions

In conclusion, we were able to identify that various factors were associated with low HGS. This study showed that low participation rate in aerobic and strengthening exercise and low educational level were associated with low HGS in the elderly Korean population. In addition, inadequate protein intake and excessive carbohydrate intake were associated with low HGS in men and alcohol drinking and diabetes were associated with low HGS in women. Further research is needed to determine if HGS improves when these relevant factors are corrected. However, implementation of relevant policies should not be limited to the low HGS group, but should consider appropriate education programs and social support for exercise and nutrition for the entire elderly population.
